# Structural dynamics insights into the M306L, M306V, and D1024N mutations in *Mycobacterium tuberculosis* inducing resistance to ethambutol

**DOI:** 10.5808/gi.23019

**Published:** 2023-09-27

**Authors:** Yustinus Maladan, Dodi Safari, Arli Aditya Parikesit

**Affiliations:** 1 Eijkman Research Center for Molecular Biology, The National Research and Innovation Agency, Cibinong, Bogor 16911, Indonesia; 2 Department of Bioinformatics, School of Life Sciences, Indonesia International Institute for Life Sciences (I3L), Jakarta 13210, Indonesia

**Keywords:** *embB* gene, ethambutol, molecular docking, *Mycobacterium tuberculosis*

## Abstract

Resistance to anti-tuberculosis drugs, especially ethambutol (EMB), has been widely reported worldwide. EMB resistance is caused by mutations in the *embB* gene, which encodes the arabinosyl transferase enzyme. This study aimed to detect mutations in the *embB* gene of *Mycobacterium tuberculosis* from Papua and to evaluate their impact on the effectiveness of EMB. We analyzed 20 samples of *M. tuberculosis* culture that had undergone whole-genome sequencing, of which 19 samples were of sufficient quality for further bioinformatics analysis. Mutation analysis was performed using TBProfiler, which identified M306L, M306V, D1024N, and E378A mutations. In sample TB035, the M306L mutation was present along with E378A. The binding affinity of EMB to arabinosyl transferase was calculated using AutoDock Vina. The molecular docking results revealed that all mutants demonstrated an increased binding affinity to EMB compared to the native protein (–0.948 kcal/mol). The presence of the M306L mutation, when coexisting with E378A, resulted in a slight increase in binding affinity compared to the M306L mutation alone. The molecular dynamics simulation results indicated that the M306L, M306L + E378A, M306V, and E378A mutants decreased protein stability. Conversely, the D1024N mutant exhibited stability comparable to the native protein. In conclusion, this study suggests that the M306L, M306L + E378A, M306V, and E378A mutations may contribute to EMB resistance, while the D1024N mutation may be consistent with continued susceptibility to EMB.

## Introduction

Tuberculosis (TB) ranks among the leading infectious diseases causing fatalities in numerous countries. The causative agent of TB is *Mycobacterium tuberculosis*. Numerous strategies have been employed to fight this disease, including the development of diagnostic tools, studying transmission patterns, understanding drug resistance, improving drug therapy, and implementing prevention and control programs. In 2019, Indonesia was among eight countries that collectively accounted for two-thirds of the global TB cases, contributing 8.5% to this total [1]. However, in 2020, there was a 14% decrease in reported TB cases from countries worldwide, including Indonesia. This decline is likely attributable to the impact of the coronavirus disease 2019 pandemic [2].

Drug-resistant *M. tuberculosis* presents a major challenge in TB control [3]. Case reports of *M. tuberculosis* being resistant to drugs such as rifampicin, isoniazid, ethambutol (EMB), streptomycin, and other anti-tuberculosis drugs have been widely reported [4,5]. Therefore, it is crucial to conduct susceptibility and sensitivity tests for these drugs prior to initiating treatment. Traditional methods for testing susceptibility and resistance to anti-TB drugs can be time-consuming. A potential solution to this issue is the application of molecular methods [1]. Whole-genome sequencing (WGS) is one of the most effective molecular techniques for enhancing TB control management [6]. WGS data can be used to identify mutations in *M. tuberculosis* genes, particularly those associated with anti-TB drug resistance [6,7]. Mutations were identified by comparing the WGS data with the genome data of *M. tuberculosis* H37Rv [8].

We have previously conducted WGS with *M. tuberculosis* samples from Papua [9]. We were able to identify resistance to both first-line and second-line anti-TB drugs with the TBProfiler [10]. In the *embB* gene for *M. tuberculosis* from Papua, we found mutations in M306L, M306L + E378A, M306V, and D1024N. Previous studies have reported different results regarding the effect of mutations at codon 306 on the *embB* gene. Several studies have shown that the M306L and M306V mutants do not cause EMB resistance [11,12]. Meanwhile, several studies have shown that the M306V mutant is present in both EMB-susceptible and EMB-resistant strains [13,14]. Other studies have shown that M306L and M306V mutants are resistant to EMB [15-17].

Computational approaches or bioinformatics are very useful in revealing the effects of mutations on protein structure and its relation to specific drug resistance [18]. Understanding the structural and dynamic levels can explain the effect of mutations in the *pncA* gene on pyrazinamide resistance [19,20]. In addition to pyrazinamide, computational methods have helped to elucidate the effect of mutations in the *inhA* gene on resistance to isoniazid [20]. However, computational explanations for *M. tuberculosis* *embB* gene mutations have not been thoroughly explored. Thus, we aimed to study the effects of M306L, M306L + E378A, M306V, and D1024N mutations on EMB resistance with a computational approach.

## Methods

### Identification of mutations in the *embB* gene in *M. tuberculosis* samples from Papua

This study analyzed WGS data from *M. tuberculosis* samples obtained from suspected TB patients in Jayapura, Papua [9]. Mutations in the *embB* gene were identified using TBProfiler [10]. The number of samples used was 20.

### Phylogenetic tree construction

A phylogenetic tree was constructed using ASA3P - Automatic Bacterial Isolate Assembly, Annotation and Analyses Pipeline (https://github.com/oschwengers/asap) [21]. The maximum-likelihood algorithm was used. Lineage and drug resistance data for phylogenetics were produced with TBProfiler using the tb-profiler collate command. Furthermore, visualization of phylogenetic trees by adding lineage and drug resistance data was conducted using the iTOL web: Interactive Tree Of Life (https://itol.embl.de/).

### *M. tuberculosis* mutant *emB* construction

The 3D structure of arabinosylindoylacetylinositol synthase in *M. tuberculosis* was obtained from the Protein Data Bank (PDB) with PDB ID: 7BVF. Chain A was used. 3D model construction due to mutation of the *M. tuberculosis* *embB* gene was performed using foldX5 software [22], which was plugged into Yasara software (http://www.yasara.org/).

### Preparation of drug molecules

The 3D structure of EMB as an antibiotic for treating TB was downloaded from the PubChem database (https://pubchem.ncbi.nlm.nih.gov/) with PubChem ID: 14052. The geometry of the ligand was optimized using Avogadro 1.1 [23] with UFF forcefield. The steepest descent algorithm was employed, with the process involving 500 steps.

### Molecular docking

Molecular docking was performed using Glide Schrodinger [24]. Prior to ligand docking, the arabinosylindoylacetylinositol synthase was optimized and minimized. The grids used were X: 165.86, Y: 151.11, and X: 176.34. The grids were determined based on the EMB grid on proteins with PDB ID: 7BVF.

### Molecular dynamics simulation

The molecular dynamics (MD) simulation was prepared using the solution builder on CHARMM GUI [25,26]. The ligand FF was parameterized using the PDB coordinate, while the CHARMM36 Force Field [27] was used for the protein. The results of protein and ligand preparation were further processed using GROMACS 2019 [28] which involved equilibration and minimization. Next, an MD simulation was carried out for 100 ns. The MD interpretation was displayed in the form of a graph of the root mean square deviation (RMSD) on the backbone, root mean square fluctuation (RMSF) on C-alpha, and solvent-accessible surface area (SASA) on the protein using Grace software.

## Results

Several mutations in the *embB* gene in *M. tuberculosis* samples from Papua were found to be associated with EMB resistance. In total, 20 samples were analyzed. The mutations in the *M. tuberculosis* *embB* gene in samples obtained from Papua were M306L, M306V, D1024N, and E378A. The M306L mutation sometimes coexisted with the E378A mutant. Several other studies have also reported other mutations in the *M. tuberculosis* *embB* gene. Some of these mutations have been validated by the World Health Organization (Table 1) [9,11,14,15,29-32].

**Fig. 1.**
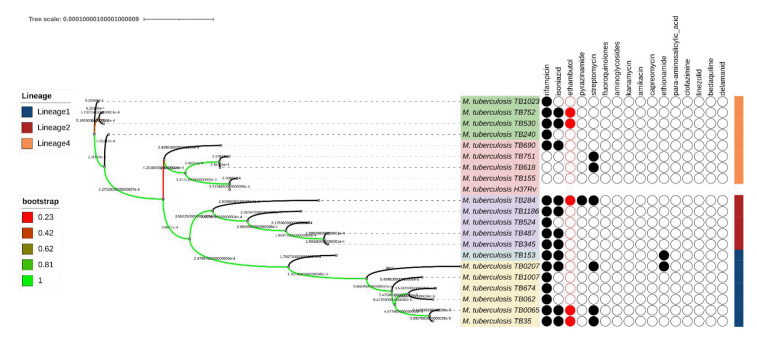
Phylogenetic tree from whole-genome sequencing results on 20 samples of *Mycobacterium tuberculosis* from Papua. Twenty samples were assigned to 5 clades. Clades I (green) and II (pitch) belonged to lineage 4, clade III (purple) originated from lineage 2, while clades IV (blue) and V (yellow) originated from lineage 1. Samples that were resistant to ethambutol were colored red, whereas resistance to other antibiotics was colored black.

The phylogenetic tree construction of 20 WGS samples of *M. tuberculosis* from Papua yielded five clades (Fig. 1). Clades I and II are groups of *M. tuberculosis* that were included in lineage 4, which is also known as the Euro-American lineage [33,34]. Clade III, with a bootstrap value of 1, was included in lineage 2, which is also known as the East Asian lineage and includes the Beijing family of strains [35,36]. Clades IV and V were members of lineage 1, commonly known as the Indo-Oceanic lineage [37] (Fig. 1). In this study, the M306L and E378A mutations were found in lineage 1, the M306V mutation in lineage 2, and the D1024N mutation in lineage 4.

**Fig. 2.**
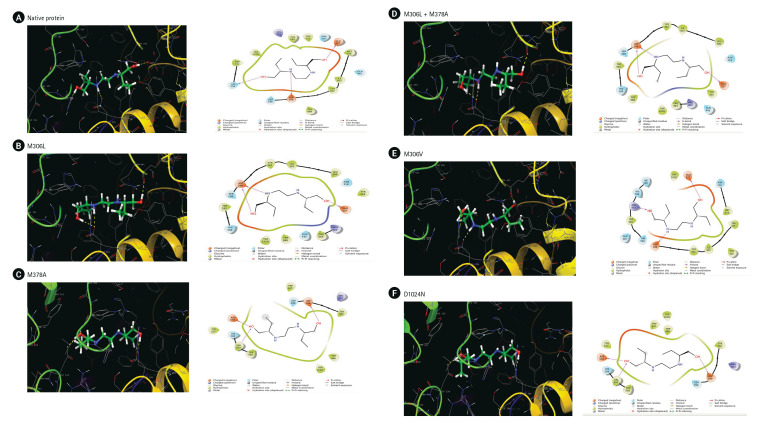
(A–F) The interaction of ethambutol with arabinosylindoylacetylinositol synthase in *Mycobaceterium tuberculosis* based on docking results.

We used molecular docking and an MD simulation to study the effects of the mutations. The docking results on the native protein revealed the formation of three hydrogen bonds at residues ASP299, Glu327, and His594 (Fig. 2A). The M306L mutant also formed three hydrogen bonds, two at the Asp299 and Glu327 residues, but it did not interact with the His594 residue as observed in the native protein (Fig. 2B). In the M378A mutant, hydrogen bonds were established at the Asp299, Asp534, and Val593 residues (Fig. 2C). When the M306L mutant was present alongside the M378A mutant, two hydrogen bonds were formed at Asp 299 and one at Glu327 (Fig. 2D). In the M306V mutant, hydrogen bonds were formed at residues Asp299, Glu327, and Arg403 (Fig. 2E). Lastly, in the D1024N mutant, hydrogen bonds were formed at Asp 299, Asp534, and Val593 (Fig. 2F).

The calculation of binding affinity, also known as the docking score, reveals that all types of mutants exhibit an increased binding affinity with EMB, as shown in Table 2. The native protein, which was used as a control, demonstrated the lowest binding energy at –0.948 kcal/mol. The M306L mutant displayed a binding affinity of –0.484 kcal/mol, which slightly increased to –0.425 kcal/mol in the presence of E378A. The M306V mutant exhibited the highest binding affinity at –0.366 kcal/mol. Conversely, the D1024 mutant showed a lesser increase in binding affinity compared to the other mutants, specifically –0.513 kcal/mol.

**Fig. 3.**
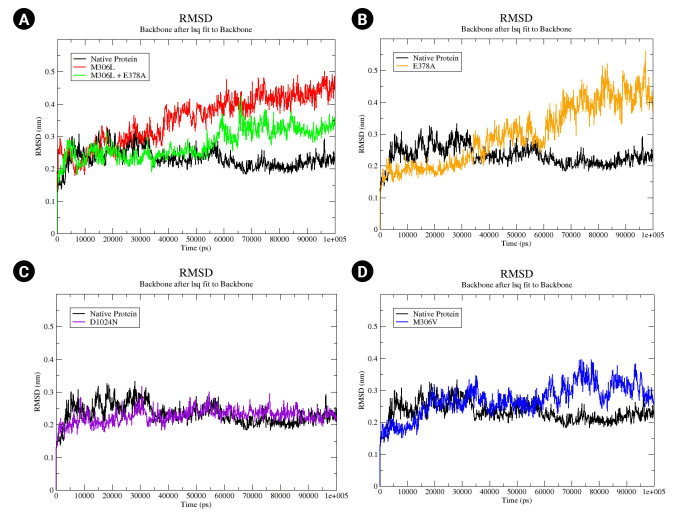
(A–D) Native root mean square deviation (RMSD) backbone protein and mutant complex during a 100,000 ps (100 ns) simulation.

An MD simulation was conducted over a period of 100,000 ps (100 ns) to observe the stability and conformational changes in arabinosylindoylacetylinositol synthase due to mutations. The parameters analyzed included RMSD, RMSF, and SASA. According to the results of the MD simulation, the native protein exhibited an RMSD of approximately 0.2 nm. In contrast, the mutants M306L, M306L + E378A, E378A, and M306V showed an increase in RMSD (Fig. 3). However, the D1024N mutant was an exception; it demonstrated a slight increase in RMSD, but returned to the same level as the native protein around the 85 ns mark (Fig. 3C). The mutants M306L and E378A experienced the most significant RMSD increase, approximately 0.4 nm (Fig. 3A and 3B). This increase in RMSD occurred around 40,000 ps (40 ns). The M306L mutant, as well as the combination of the M306L and E378A mutants, saw an RMSD increase around 60,000 ps (60 ns), with an RMSD value of 0.3 nm (Fig. 3A). The M306V mutant also experienced an RMSD increase at 60 ns (Fig. 3D). The increase in RMSD in the mutants, with the exception of the D1024N mutant, suggests instability in the mutant protein when interacting with EMB.

**Fig. 4.**
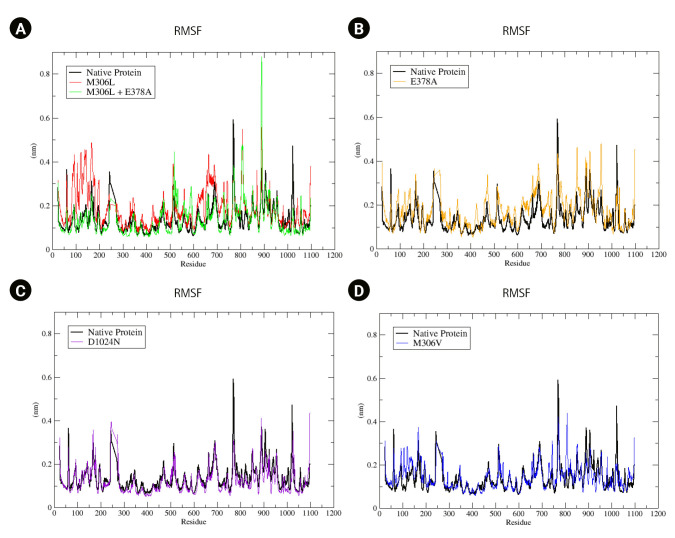
(A–D) Root mean square fluctuation (RMSF) native protein and mutant complex during a 100,000 ps (100 ns) simulation.

We also analyzed the flexibility of the EMB-protein complex in both the native protein and its mutants (Fig. 4). RMSF represents the root mean square displacement of each amino acid residue in the protein relative to the average conformation. A higher residual fluctuation signifies increased residual flexibility under pressure treatment [38]. Among the mutants, M306L and E378A exhibited the most significant residual fluctuations (Fig. 4A and 4B). Conversely, the D1024N mutant showed only a minor difference compared to the native protein (Fig. 4C). The RMSF value of the M306V mutant also showed an increase relative to the native protein (Fig. 4D). This heightened residual fluctuation suggests that the mutant protein may be less stable when bound to EMB.

**Fig. 5.**
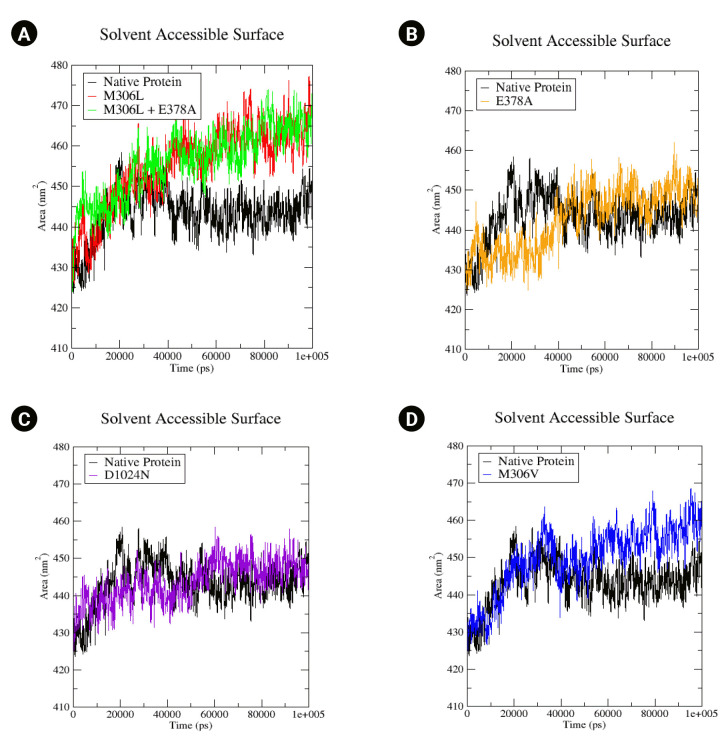
(A–D) Solvent-accessible surface of the native protein and mutant complex during a 100,000 ps (100 ns) simulation.

The proteins’ SASA values were computed. A rise in the SASA value could suggest a relative expansion [39]. In the native protein, the SASA value begins at 340 430 nm^2^, peaking at 458 nm^2^ at 20 ns, before decreasing to 440 nm^2^ by the end of the simulation (Fig. 5A). The SASA values for all mutants commenced at the same relative value as the native protein (430 nm^2^). The M306L and M306L + E378A mutants exhibited a similar increase in SASA values, reaching up to 465 nm^2^ (Fig. 5A). The M306V mutant also demonstrated an increase in SASA, up to 460 nm^2^ (Fig. 5D). The E378A mutant experienced a rise in SASA values as well, up to 450 nm^2^ (Fig. 5B). Conversely, the D1024N mutant only showed a minor increase in the SASA value (approximately 445 nm^2^) (Fig. 5C). An increase in the SASA value signifies enhanced access to the mutant surface compared to the native protein.

## Discussion

The *embB* gene of *M. tuberculosis* produces arabinosylindoylacetylinositol synthase. This enzyme plays a role in the biosynthesis of arabinan mycobacterial cell walls. Certain mutations in the *embB* gene can cause resistance to EMB. In this study we used a computational approach to elucidate the effect of multiple mutations in the *M. tuberculosis* *embB* gene on EMB resistance, using currently available 3D structural data of arabinosylindoylacetylinositol synthase in *M. tuberculosis* H37Rv (PDB ID: 7BVF). This structure provides a valuable resource for studying various aspects related to the function of arabinosylindoylacetylinositol synthase, including the influence of *embB* gene mutations on EMB resistance.

In the M306L mutant, molecular docking results indicated an enhanced binding affinity to EMB. When the M306L mutant was present concurrently with E378A, there was a marginal increase in binding affinity compared to the single condition of the M306L mutant (Table 2). The M306V mutant also demonstrated an increased binding affinity to EMB. The MD simulation results were consistent with the molecular docking findings, as evidenced by the increased RMSD value (Fig. 3) and RMSF (Fig. 4) during the simulation.

The increase in the RMSD and RMSF values of the mutants suggested instability in the mutant protein. This instability in protein structure could potentially influence EMB resistance. This finding aligns with previous studies, which have shown that the M306L, M306V, and E378A mutants contribute to EMB resistance [15-17,29]. According to the World Health Organization database, the M306L and M306V mutants are indeed associated with EMB resistance. However, the same database indicates that the E378A mutation is not linked to EMB resistance [30].

The D1024N mutant demonstrated an increased binding affinity to EMB, although not to the same extent as other mutants (Table 2). The RMSD value for the D1024N mutant showed a slight increase compared to the native protein (Fig. 3C). Similarly, the RMSF and SASA tilapia values were slightly higher than those of the native protein. This suggests that the D1024N mutation does not significantly impact the stability of the protein. Therefore, the computational data for the D1024N mutant suggests that it may not significantly affect EMB or potentially cause resistance to EMB. This is consistent with the results of drug susceptibility testing for the D1024N mutant, which indicated low-level EMB resistance [31]. Another study found that samples containing the D1024N mutation did not result in EMB resistance [15]. According to the World Health Organization database, the D1024N mutant is currently classified as being of "uncertain significance."

The results of our computational analysis provide a clear explanation of the effects of the M306L, M306L + E378A, M306V, and D1024N mutations. The M306L, M306V, and E378A mutations appear to impact the stability of arabinosylindolylacetylinositol synthase, which could potentially lead to resistance to EMB. Conversely, the D1024N mutation does not seem to affect protein stability, suggesting that this mutation may not result in EMB resistance.

## Figures and Tables

**Table 1.** Mutations in the *embB* gene in *Mycobacterium tuberculosis* samples from Papua and several other mutations

Codon	Amino acid change	Reference	World Health Organization [30]
306	Methionine → leucine*, valine*, isoleucine	[9,15,32]	R
328	Aspartic acid → tyrosine	[29]	R
378	Glutamic acid → alanine*	[9,29]	S
354	Aspartic acid → alanine	[11]	R
406	Glycine → alanine, aspartic acid	[11,29]	R
497	Glutamine → arginine	[14]	R
1024	Aspartic acid → asparagine*, threonine	[9,29,31]	Uncertain significance

Mutations found in Papua are marked with an asterisk.

**Table 2.** Docking scores

No.	Mutation	Docking score (kcal/mol)	Glide model
1	Native	–0.948	–30.764
2	M306L	–0.484	–28.823
3	M306L + E378A	–0.425	–27.373
4	E378A	–0.463	–28.779
5	M306V	–0.366	–26.504
6	D1024N	–0.513	–29.146
			

## References

[1] (2020). Global tuberculosis report 2020. https://apps.who.int/iris/bitstream/handle/10665/336069/9789240013131-eng.pdf.

[2] (2021). Global tuberculosis report 2021. https://www.who.int/publications/i/item/9789240037021.

[3] (2016). Global leprosy strategy 2016-2020: accelerating towards a leprosy-free world. Vol. 1, Weekly epidemiological record. http://apps.who.int/iris/bitstream/10665/205149/1/B5233.pdf?ua=1.

[4] Brown AC, Bryant JM, Einer-Jensen K, Holdstock J, Houniet DT, Chan JZ (2015). Rapid whole-genome sequencing of *Mycobacterium tuberculosis* isolates directly from clinical samples. J Clin Microbiol.

[5] Dixit A, Freschi L, Vargas R, Calderon R, Sacchettini J, Drobniewski F (2019). Whole genome sequencing identifies bacterial factors affecting transmission of multidrug-resistant tuberculosis in a high-prevalence setting. Sci Rep.

[6] World Health Organization (2018). The Use of Next-Generation Sequencing Technologies for the Detection of Mutations Associated with Drug Resistance in *Mycobacterium tuberculosis* Complex: Technical Guide.

[7] Coll F, McNerney R, Preston MD, Guerra-Assuncao JA, Warry A, Hill-Cawthorne G (2015). Rapid determination of anti-tuberculosis drug resistance from whole-genome sequences. Genome Med.

[8] Cole ST, Brosch R, Parkhill J, Garnier T, Churcher C, Harris D (1998). Deciphering the biology of *Mycobacterium tuberculosis* from the complete genome sequence. Nature.

[9] Maladan Y, Krismawati H, Wahyuni T, Tanjung R, Awaludin K, Audah KA (2021). The whole-genome sequencing in predicting *Mycobacterium tuberculosis* drug susceptibility and resistance in Papua, Indonesia. BMC Genomics.

[10] Phelan JE, O'Sullivan DM, Machado D, Ramos J, Oppong YE, Campino S (2019). Integrating informatics tools and portable sequencing technology for rapid detection of resistance to anti-tuberculous drugs. Genome Med.

[11] Ruesen C, Riza AL, Florescu A, Chaidir L, Editoiu C, Aalders N (2018). Linking minimum inhibitory concentrations to whole genome sequence-predicted drug resistance in *Mycobacterium tuberculosis* strains from Romania. Sci Rep.

[12] Hazbon MH, Bobadilla del Valle M, Guerrero MI, Varma-Basil M, Filliol I, Cavatore M (2005). Role of *embB* codon 306 mutations in *Mycobacterium tuberculosis* revisited: a novel association with broad drug resistance and IS6110 clustering rather than ethambutol resistance. Antimicrob Agents Chemother.

[13] Bakula Z, Napiorkowska A, Bielecki J, Augustynowicz-Kopec E, Zwolska Z, Jagielski T (2013). Mutations in the *embB* gene and their association with ethambutol resistance in multidrug-resistant *Mycobacterium tuberculosis* clinical isolates from Poland. Biomed Res Int.

[14] Li MC, Chen R, Lin SQ, Lu Y, Liu HC, Li GL (2020). Detecting ethambutol resistance in *Mycobacterium tuberculosis* isolates in China: a comparison between phenotypic drug susceptibility testing methods and DNA sequencing of embAB. Front Microbiol.

[15] Sekiguchi J, Miyoshi-Akiyama T, Augustynowicz-Kopec E, Zwolska Z, Kirikae F, Toyota E (2007). Detection of multidrug resistance in *Mycobacterium tuberculosis*. J Clin Microbiol.

[16] Sreevatsan S, Stockbauer KE, Pan X, Kreiswirth BN, Moghazeh SL, Jacobs WR (1997). Ethambutol resistance in *Mycobacterium tuberculosis*: critical role of *embB* mutations. Antimicrob Agents Chemother.

[17] Lee AS, Othman SN, Ho YM, Wong SY (2004). Novel mutations within the *embB* gene in ethambutol-susceptible clinical isolates of *Mycobacterium tuberculosis*. Antimicrob Agents Chemother.

[18] Kumar S, Jena L (2014). Understanding rifampicin resistance in tuberculosis through a computational approach. Genomics Inform.

[19] Alatawi EA, Alshabrmi FM (2022). Structural and dynamic insights into the W68L, L85P, and T87A mutations of *Mycobacterium tuberculosis* inducing resistance to pyrazinamide. Int J Environ Res Public Health.

[20] Kumar V, Sobhia ME (2015). Molecular dynamics assisted mechanistic study of isoniazid-resistance against *Mycobacterium tuberculosis* InhA. PLoS One.

[21] Schwengers O, Hoek A, Fritzenwanker M, Falgenhauer L, Hain T, Chakraborty T (2020). ASA3P: an automatic and scalable pipeline for the assembly, annotation and higher-level analysis of closely related bacterial isolates. PLoS Comput Biol.

[22] Schymkowitz J, Borg J, Stricher F, Nys R, Rousseau F, Serrano L (2005). The FoldX web server: an online force field. Nucleic Acids Res.

[23] Hanwell MD, Curtis DE, Lonie DC, Vandermeersch T, Zurek E, Hutchison GR (2012). Avogadro: an advanced semantic chemical editor, visualization, and analysis platform. J Cheminform.

[24] Friesner RA, Banks JL, Murphy RB, Halgren TA, Klicic JJ, Mainz DT (2004). Glide: a new approach for rapid, accurate docking and scoring. 1. Method and assessment of docking accuracy. J Med Chem.

[25] Jo S, Kim T, Iyer VG, Im W (2008). CHARMM-GUI: a web-based graphical user interface for CHARMM. J Comput Chem.

[26] Lee J, Cheng X, Swails JM, Yeom MS, Eastman PK, Lemkul JA (2016). CHARMM-GUI Input Generator for NAMD, GROMACS, AMBER, OpenMM, and CHARMM/OpenMM simulations using the CHARMM36 additive force field. J Chem Theory Comput.

[27] Huang J, Rauscher S, Nawrocki G, Ran T, Feig M, de Groot BL (2017). CHARMM36m: an improved force field for folded and intrinsically disordered proteins. Nat Methods.

[28] Abraham MJ, Murtola T, Schulz R, Pall S, Smith JC, Hess B (2015). GROMACS: high performance molecular simulations through multi-level parallelism from laptops to supercomputers. SoftwareX.

[29] Ali A, Hasan Z, McNerney R, Mallard K, Hill-Cawthorne G, Coll F (2015). Whole genome sequencing based characterization of extensively drug-resistant *Mycobacterium tuberculosis* isolates from Pakistan. PLoS One.

[30] World Health Organization (2021). Catalogue of mutations in *Mycobacterium tuberculosis* complex and their association with drug resistance.

[31] Eldholm V, Norheim G, von der Lippe B, Kinander W, Dahle UR, Caugant DA (2014). Evolution of extensively drug-resistant *Mycobacterium tuberculosis* from a susceptible ancestor in a single patient. Genome Biol.

[32] Mokrousov I, Otten T, Vyshnevskiy B, Narvskaya O (2002). Detection of *embB*306 mutations in ethambutol-susceptible clinical isolates of *Mycobacterium tuberculosis* from Northwestern Russia: implications for genotypic resistance testing. J Clin Microbiol.

[33] Senghore M, Diarra B, Gehre F, Otu J, Worwui A, Muhammad AK (2020). Evolution of *Mycobacterium tuberculosis* complex lineages and their role in an emerging threat of multidrug resistant tuberculosis in Bamako, Mali. Sci Rep.

[34] Gagneux S (2012). Host-pathogen coevolution in human tuberculosis. Philos Trans R Soc Lond B Biol Sci.

[35] Coscolla M, Gagneux S (2014). Consequences of genomic diversity in *Mycobacterium tuberculosis*. Semin Immunol.

[36] Kato-Maeda M, Shanley CA, Ackart D, Jarlsberg LG, Shang S, Obregon-Henao A (2012). Beijing sublineages of *Mycobacterium tuberculosis* differ in pathogenicity in the guinea pig. Clin Vaccine Immunol.

[37] Abdelhaleem A, Hershan A, Agarwal P, Farasani A, Omar SV, Ismail A (2020). Whole-genome sequencing of a *Mycobacterium tuberculosis* strain belonging to lineage 1 (Indo-Oceanic) and the East African Indian spoligotype, isolated in Jazan, Saudi Arabia. Microbiol Resour Announc.

[38] Huang Y, Zhang X, Suo H (2021). Interaction between beta-lactoglobulin and EGCG under high-pressure by molecular dynamics simulation. PLoS One.

[39] Krebs BB, De Mesquita JF (2016). Amyotrophic lateral sclerosis type 20: in silico analysis and molecular dynamics simulation of hnRNPA1. PLoS One.

